# Simultaneous versus sequential fractional CO_2_ laser and subcision combination for management of post‐acne atrophic scars: A split‐face comparative study

**DOI:** 10.1111/jocd.16395

**Published:** 2024-05-21

**Authors:** Bao Quoc Tran, Tu Nguyen Anh Tran, Em Van Loi Doan, Thuy Thi Phan Nguyen, Hao Trong Nguyen

**Affiliations:** ^1^ Department of Dermatology Ho Chi Minh City Hospital of Dermato‐Venereology Ho Chi Minh City Vietnam; ^2^ Department of Dermatology Pham Ngoc Thach University of Medicine Ho Chi Minh City Vietnam

**Keywords:** fractional CO_2_ laser, post‐acne atrophic scars, sequential, simultaneous, subcision

## Abstract

**Background:**

A combination of fractional CO_2_ laser and subcision is usually employed for the treatment of post‐acne atrophic scars. However, the efficacy and safety of both simultaneous and sequential combination therapies should be explored.

**Aims:**

To compare the efficacy and safety of simultaneous and sequential fractional CO_2_ laser and subcision combination therapies for post‐acne atrophic scars.

**Patients and Methods:**

This single‐blind, split‐face clinical trial included 34 patients with post‐acne atrophic scars at our institution. Each patient underwent three sessions of subcision combined with fractional CO_2_ laser, with a 1‐month interval between each session. The left side of the face was treated with simultaneous combination therapy, whereas the right side was treated with sequential combination therapy. Treatment efficacy was assessed at 4, 8, and 12 weeks; and 3 and 6 months after the last session.

**Results:**

Simultaneous and sequential treatments demonstrated comparable efficacy. Regarding the adverse events, the side of the face undergoing simultaneous treatment experienced longer swelling duration, higher pain levels during laser treatment, and shorter downtime.

**Conclusions:**

Despite the longer swelling time and higher pain levels during laser treatment in the simultaneous treatment side, the effectiveness and satisfaction level of the CO_2_ fractional laser and subcision for treatment of the acne scars were comparable between the two combinations, with a shorter downtime for the simultaneous than for the sequential combination therapy.

## 1 INTRODUCTION

Acne vulgaris is a disease of the pilosebaceous unit affecting 9.4% of the global population.^1^ Abnormal wound healing may result in the development of atrophic scars in 95% of patients with acne.^2^ Scars are cosmetically unattractive and cause severe psychosocial discomfort. Management of atrophic acne scars is complex owing to the absence of a gold standard treatment; thus, combined treatment modalities are employed for the management of atrophic acne scars.^3^, ^4^ Fractional CO_2_ lasers and subcision are effective methods for treating post‐acne atrophic scars. Fractional CO_2_ lasers stimulate collagen synthesis through microthermal zones, whereas subcision mechanically separates scars from the underlying tissues. However, the efficacy and safety of these treatments when applied simultaneously versus sequentially have not been elucidated.

This study aimed to fill this knowledge gap by evaluating the efficacy and safety of simultaneous and sequential fractional CO_2_ laser and subcision treatments using a split‐face comparative design. We hypothesized that uninterrupted wound healing during simultaneous fractional CO_2_ laser treatment may result in improved cosmetic and clinical outcomes. We assessed the treatment efficacy using the echelle d'evaluation clinique des cicatrices d'acne (ECCA) score, Investigators Global Assessment scale, patient satisfaction, and skin thickness measurements using high‐frequency ultrasound. Thus, we aimed to develop effective and patient‐centric treatment strategies for the management of post‐acne atrophic scars.

## 2 PATIENTS AND METHODS

### 2.1 Patient selection

Thirty‐four patients with post‐acne atrophic scars on both sides of their faces were enrolled from the Department of Cosmetic Dermatology at our institution. The recruitment period was from October 2022 to June 2023.

Pregnant or lactating women; patients who had used oral isotretinoin within the previous 6 months or topical retinoids within the last 2 weeks; patients receiving anticoagulative or antiplatelet medications or immunosuppressive therapy; and those who had active moderate‐to‐severe acne vulgaris, active infections (including Herpes simplex infection) at the treatment sites, undergone specific cosmetic procedures within the last 6 months, and systemic diseases or a history of keloids were excluded from the study.

### 2.2 Study design

Each patient underwent three sessions of subcision combined with fractional CO_2_ laser, with a 1‐month interval between each session. The left side of the face was treated with simultaneous combination therapy, in which fractional CO_2_ laser therapy was performed immediately after subcision. The right side of the face received sequential combination therapy with a fractional CO_2_ laser 2 weeks after subcision.

### 2.3 Before the procedure

Participants with a history of herpes simplex infection were prescribed oral antiviral tablets containing 400 mg acyclovir twice daily for 5 days, starting 2 days before the procedure. Preoperative lidocaine 2.5% and prilocaine 2.5% cream (EMLA, a eutectic mixture of local anesthetic; AstraZeneca LP, Wilmington, DE, USA) was applied under occlusion for 60 min before the removal of the local anesthetic. A 500 mg paracetamol‐based oral analgesic was administered immediately before the procedure.

### 2.4 Fractional CO_2_ laser

All the participants underwent fractional CO_2_ laser (10600‐nm eCO_2_ laser, Lutronic Corporation, Goyang, Korea) therapy on both sides of the face sequentially with a 4‐week interval between the sessions for each side of the face by a single dermatologist with the following parameters at static mode: 120 μm beam size, 30 W peak power, 50–75 spots/cm^2^ density, with two passes. In the first pass, only the atrophic scars were treated using a 4‐mm square or circle pattern, 100–120 mJ pulse energy, and 50 spots/cm^2^ density in the static operating mode. In the second pass, the entire half‐side of the face was treated using a 12‐mm square pattern, 30–40 mJ pulse energy, and 75 spots/cm^2^ density in the static operating mode with less than 10% overlap.

### 2.5 Subcision

Subcision was performed in all the participants using a 181/2G Nokor needle (BD Nokor™ Admix Needles, Becton, Dickinson and Company, New Jersey, USA). The surgical sites were sterilized with alcohol pads and intradermally injected with a 2% lidocaine solution. After attaining an appropriate level of local anesthesia, a Nokor needle was inserted into the skin close to the depressed scars until it lay precisely under the scar. For several small to medium‐sized scars close to each other, subcision was performed through a single entry point to reduce bleeding and minimize unnecessary skin damage. For larger scars, those varied in depth or those spaced further apart, subcision was performed individually for each scar. The insertion depth was maintained at the dermal‐hypodermal junction. The subcision needle was initially advanced and retracted in a tunneling motion to penetrate the fibrotic scar tissue. Once the fibrous mass was sufficiently fractured, the needle was slid parallel to the surface of the skin to detach the scar from its tethers. Several needle insertion sites were used to undermine defects from various angles for densely fibrous scars.

Subcision was performed immediately before fractional CO_2_ laser session in simultaneous treatment, and 2 weeks prior to fractional CO_2_ laser session in sequential treatment.

### 2.6 Clinical evaluation and follow‐up

Photographs (five photos taken at 0°, 45°, and 90° to each half of the face and multiple close‐ups at the scar sites) were taken at baseline, before each treatment session, and at the first (1 month), second (3 months), and third (6 months) follow‐ups after the last session. The head position, distance from the camera, and illumination was kept constant when comparing the images taken before and after therapy. The global response to treatment was evaluated by a blinded investigator using patient photographs before and after treatment. The rating of the investigator's Global Response to treatment was performed using a quartile grading scale (0 = no improvement, 0%; 1 = mild improvement, 1%–25%; 2 = moderate improvement 25%–49%; 3 = significant improvement 50%–74%; and 4 = marked improvement ≥75%). The ECCA score provides a quantitative grading tool for evaluating the severity and clinical improvement of acne scars.^5^

### 2.7 Adverse effects

Adverse effects, such as pain (using a 10‐point visual analog scale), downtime, duration of erythema, swelling, crusting, bruising, incidence of post‐inflammatory hyperpigmentation (PIH), acne, and other side effects, were documented for each side of the face. The patients were asked to evaluate the side effects at each session and at the first and second follow‐ups.

### 2.8 Patient satisfaction

Patients were asked to rate their satisfaction with each type of combination therapy on a four‐point Likert scale according to the following categories: extremely satisfied, satisfied, partially satisfied, and dissatisfied.

### 2.9 High‐frequency ultrasonographic assessment

Before treatment and 3 months after the final session, a high‐frequency ultrasound system (HFUS, Supersonic Mach 30, Hologic, SuperSonic Imagine, Aix‐en‐Provence, France), which uses a 20 MHz linear probe with 8‐mm penetration, 30 × 12 m μm resolution, provided quantitative assessment and measurements in mm. The acquired HFUS images were imported into MATLAB's image segmenter to segment and delineate the skin layers. A MATLAB code was applied to assess the skin thickness from the segmented images. The overall thickness of the skin, epidermis, and dermis at the treatment sites were also measured (mm).

### 2.10 Statistical analysis

Statistical analyses were performed using the IBM SPSS Statistics 26. Continuous variables with normal distributions are presented as means ± standard deviation, while those with abnormal distributions are presented as medians (interquartile ranges). Categorical variables are summarized as frequencies and percentages. Student's *t*‐test and the Mann–Whitney‐Wilcoxon test was used to analyze the differences between dependent numerical groups with and without a normal distribution, respectively. A paired *t*‐test or Wilcoxon signed‐rank test was used to compare continuous variables within the same group. For dichotomous variables, the Chi‐squared test or Fisher's exact test was used, as considered appropriate. Statistical significance was set at *p* < 0.05 is considered significant. Generalized estimating equations (GEE) were used to analyze correlated continuous variables with repeated measurements over time, using a linear model for variables with normal distribution and gamma with log link for variables without normal distribution.

## 3 RESULTS

### 3.1 Patient demographics

The study comprised 34 patients, 24 males (70.6%) and 10 females (29.4%), with an age range of 23 to 41 years and a mean age of 30.38 ± 4.34 years. The age of onset of the acne scar was 17.97 ± 3.83 years, ranging from 11 to 27 years. The average duration of the condition was 12.41 ± 5.49 years, with the shortest being 2 years and the most extended 23 years. The predominant type of scarring varied among the patients, with mixed scars being the most common (47.1%). The Fitzpatrick skin types ranged from type 2 to 5, with types 3 (41.2%) and 4 (35.3%) being the most prevalent. The baseline ECCA scores were 128 ± 31.81 and 125 ± 35.12 on the left and right sides of the face, respectively, with no statistically significant difference between the two sides. Pre‐treatment skin thickness measurements revealed that the epidermis, dermis, and total skin thicknesses were 0.3 mm, 1.26 mm, and 1.56 mm, respectively on the left side of the face, and 0.29 mm, 1.29 mm, and 1.58 mm, respectively on the right side, with no statistically significant differences between the two sides.

### 3.2 Clinical assessment

The global response to treatment at multiple time points during treatment and 6 months after the last treatment (Week 32) demonstrated no statistically significant differences between the level of improvement on either side when evaluated by a blinded investigator (*p* = 0.687) (*p* = 0.687). When evaluated by a blinded physician at 6 months on the simultaneous side, 3 (8.8%), 17 (50%), 9 (26.47%), and 5 (14.7%) patients showed mild, moderate, marked, and significant improvement in their acne scars, respectively. Comparatively, the sequential side exhibited a similar pattern. Mild improvement was observed in three (8.82%) patients. Most patients (15, [44.12%]) demonstrated moderate improvement. Significant and marked improvements were observed in 11 (32.35%) and 5 (14.7%) patients, respectively. (Figure 1).

**FIGURE 1:**
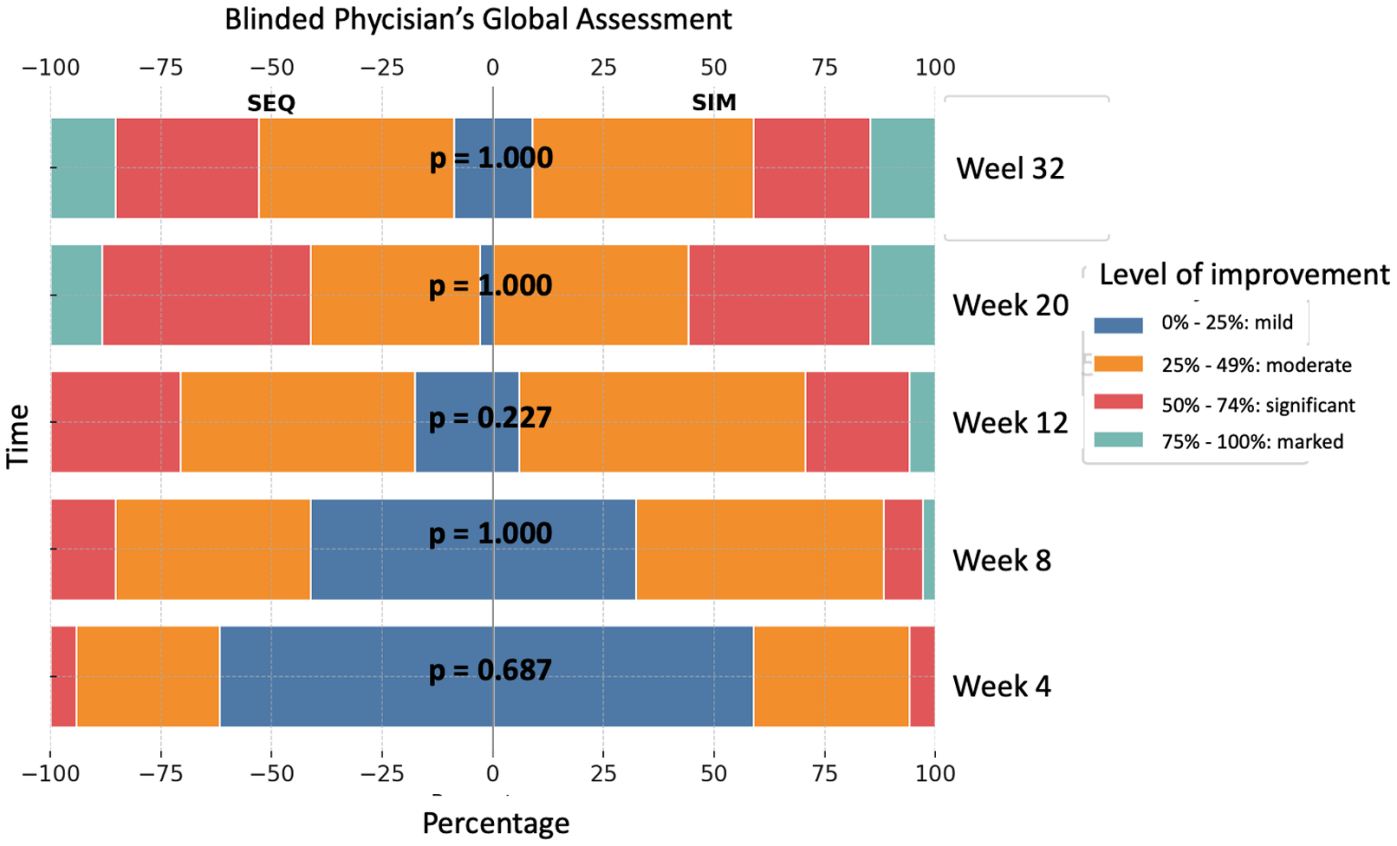
Blinded physician's global assessment.

The ECCA scores in the simultaneous side decreased from 128.24 ± 31.81 to 94.71 ± 34.16 with a 26.15% improvement, while the sequential side decreased from 124.71 ± 35.12 to 99.41 ± 37.19, demonstrating a 20.29% improvement. The ECCA scores were analyzed using GEE with a gamma distribution and a log link function, accounting for the within‐subject correlation of repeated measurements. The model incorporated “subject” as a random effect and both “treatment” and “time” as fixed effects. A significant time effect was observed (Wald Chi‐squared = 127.857, df = 5, *p* < 0.001), indicating that ECCA scores varied significantly over the six time points, regardless of the treatment side. This effect was consistent across all the time points except for that for Week 20 compared with Week 32, indicating that the increase was statistically insignificant. The analysis did not reveal a significant effect of treatment on the ECCA scores (Wald Chi‐squared = 0.142, df = 1, *p* = 0.706). This suggests that the ECCA scores were not significantly different between the sequential and simultaneous treatment sides. No significant interaction was observed between the time and treatment (Wald Chi‐squared = 6.718, df = 5, *p* = 0.242), indicating that the effect of treatment on the ECCA scores did not vary significantly across different time points, and that the efficacy of the two treatments was comparable (Figures 2, 3, 4; Table 1).

**FIGURE 2:**
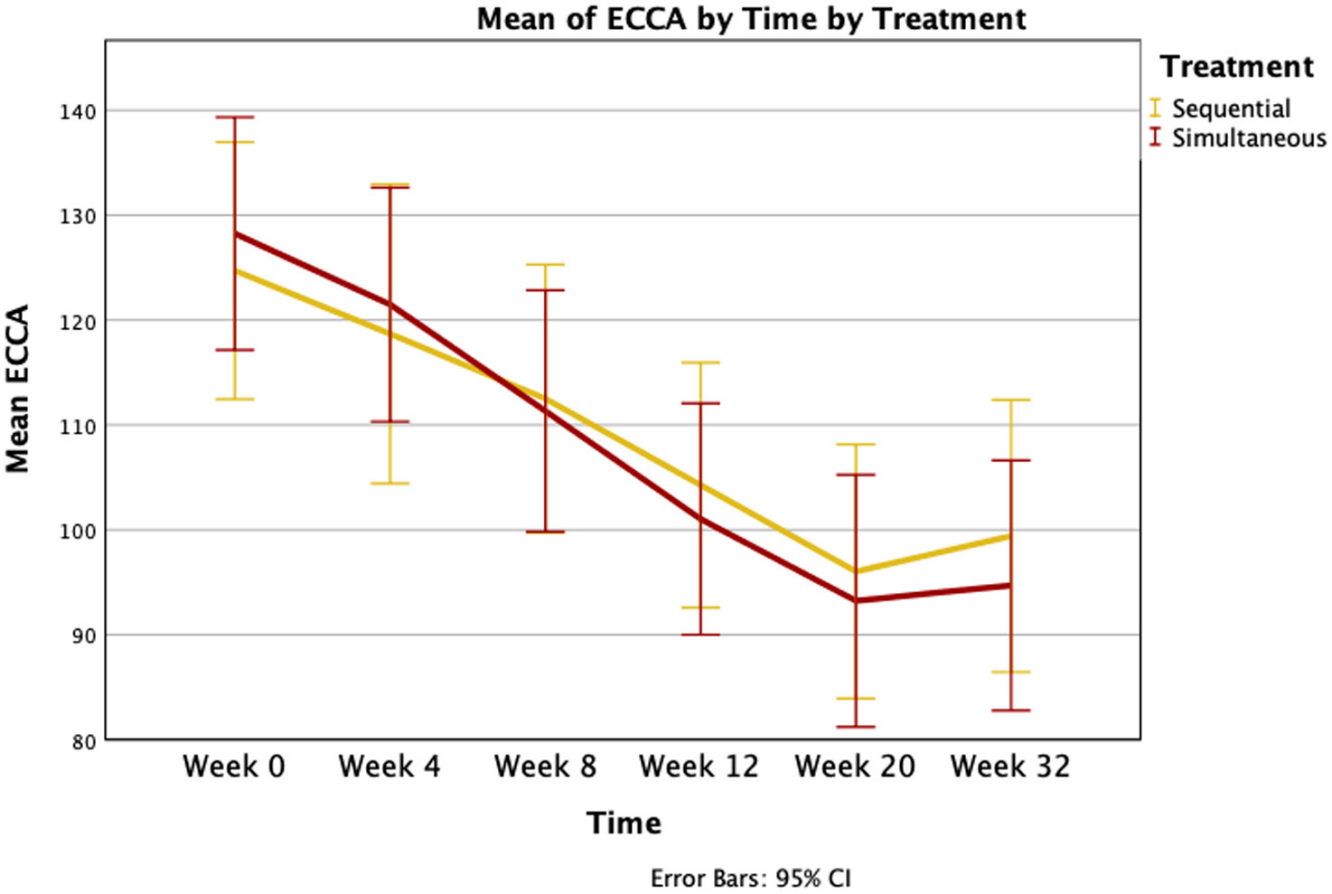
ECCA score by time and treatment.

**FIGURE 3:**
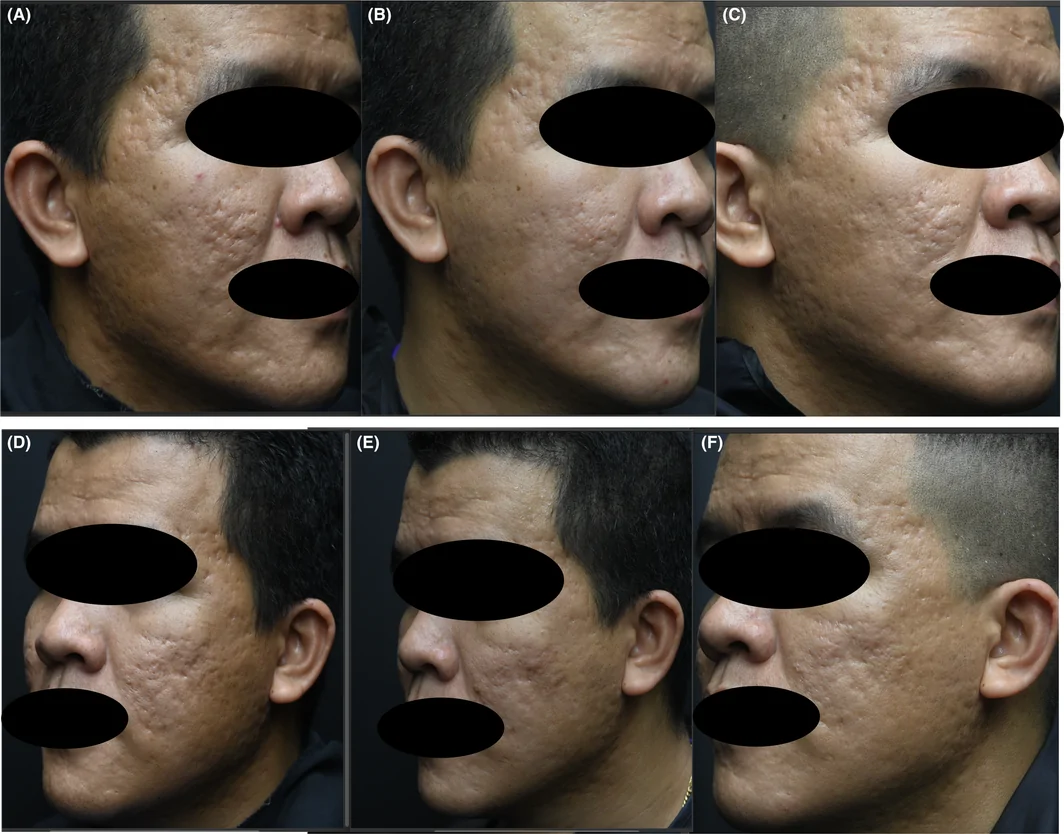
A case of a 29‐year‐old male patient with multiple rolling, icepick, and boxcar scars. The patient underwent full‐face subcision; fractional CO_2_ laser resurfacing was performed immediately (simultaneous side) at the left side. Fractional CO_2_ laser resurfacing was performed 2 weeks after subcision (sequential side) at the right side. This combined treatment was repeated for a total of three sessions, 4 weeks apart. (A) Right side before treatment. (D) Left side before treatment. (B) Right side 3 months after last session. (E) Left side 3 months after last session. (C) Right side 6 months after last session. (F) Right side 6 months after last session.

**FIGURE 4:**
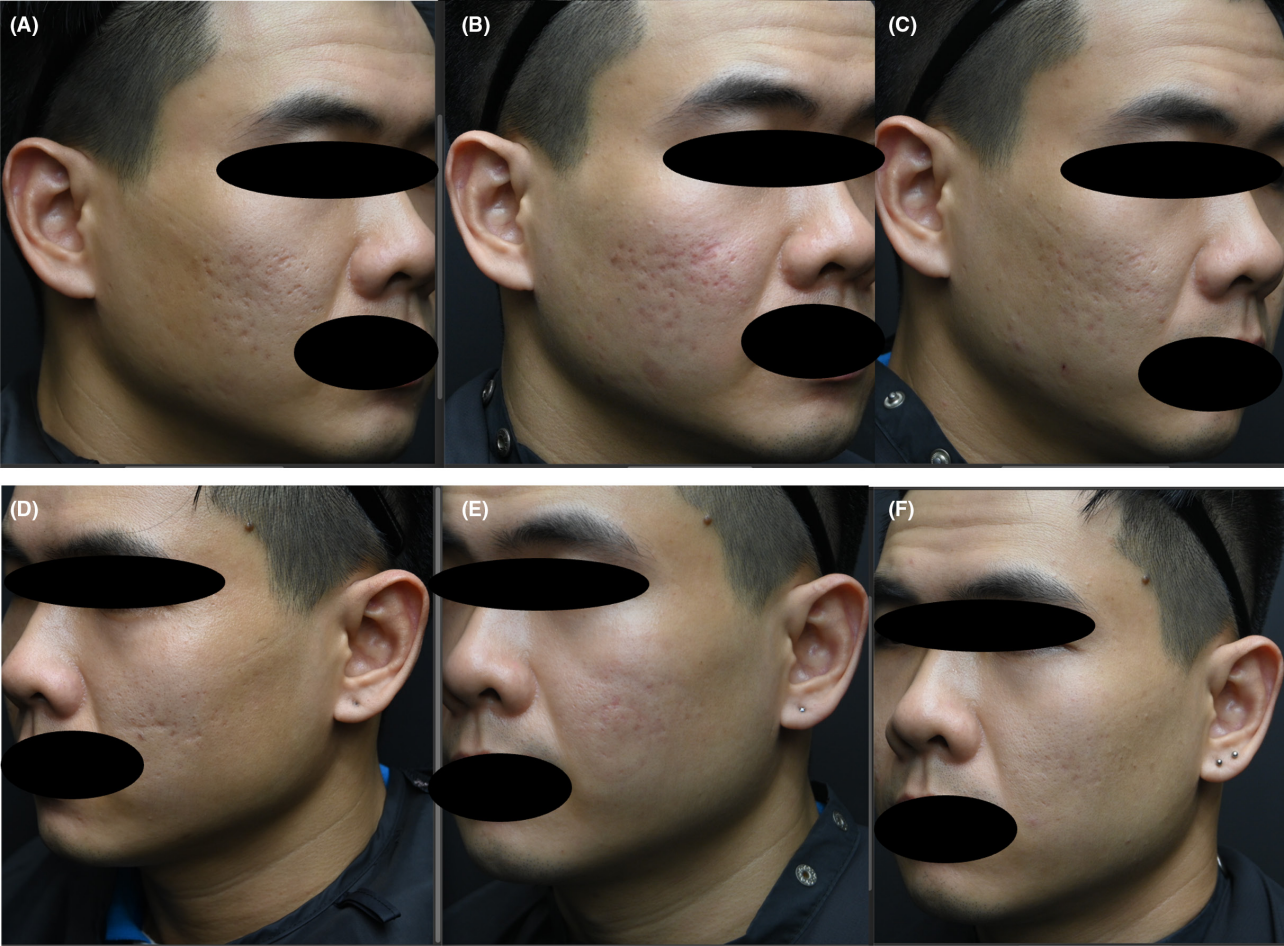
A case of a 32‐year‐old male patient with multiple icepick and boxcar scars. The patient underwent full‐face subcision; fractional CO2 laser resurfacing was performed immediately (simultaneous side) at the left side. Fractional CO2 laser resurfacing was performed 2 weeks after subcision (sequential side) at the right side. This combined treatment was repeated for a total of three sessions, 4 weeks apart. (A) Right side before treatment. (D) Left side before treatment. (B) Right side 3 months after last session. (E) Left side 3 months after last session. (C) Right side 6 months after last session. (F) Right side 6 months after last session.

**TABLE 1:** Generalized estimating equations result of the ECCA score.

Tests of model effects			
Source	Type III		
	Wald Chi‐square	Df	*p*‐value
(Intercept)	8813.507	1	0.000
Time	127.857	5	0.000
Treatment	0.142	1	0.706
Time × Treatment	6.718	5	0.242
Dependent variable: ECCA Model: (Intercept), Time, Treatment, Time × Treatment			

Abbreviations: Df, degrees of freedom; ECCA, echelle d'evaluation clinique des cicatrices d'acne.

### 3.3 Patient satisfaction

Regarding patient satisfaction using a quartile scale, no significant difference was observed between the two sides after treatment (*p* = 1.000). The largest proportion of patients (58.5%) reported being “satisfied” considering both treatment sides. The second largest category for both treatments was “partially satisfied,” with 20.6% of the responses. The “extremely satisfied” group represented 17.6% of the responses for both sides. The smallest group for both treatments was “dissatisfied,” with 2.9% of the responses.

### 3.4 High‐frequency ultrasonographic assessment

The thickness of the epidermis and dermis and the total skin thickness were obtained using HFUS at baseline (Week 0), 3 months (Week 20), and 6 months (Week 32) after the last treatment session. The thickness of the epidermis for the simultaneous and sequential sides at Week 0 was 0.3 mm ± 0.06 and 0.29 mm (0.04), respectively. At Week 20, these values were 0.29 mm ± 0.06 for the simultaneous side and 0.27 mm (0.08) for the sequential side. Finally, at Week 32, the thickness was 0.29 mm ± 0.05 and 0.3 mm (0.06) for the simultaneous and sequential side, respectively. GEE analysis revealed no significant effects of treatment (*p* = 0.954), time (*p* = 0.495), or interaction between treatment and time point (*p* = 0.701) on the epidermal thickness.

For the dermis, the baseline thickness was 1.26 mm (0.25) for the simultaneous side and 1.29 mm (0.24) for the sequential side. These values changed to 1.4 mm (0.63) and 1.55 mm (0.49) at Week 20, and were 1.52 mm (0.41) and 1.48 mm (0.33) at Week 32, respectively. GEE analysis revealed significant time point effects (*p* < 0.001) but no significant treatment effect (*p* = 0.781) or treatment‐time interaction (*p* = 0.491).

Regarding the total skin thickness, the initial thickness was 1.56 mm (0.27) and 1.58 mm (0.27) for the simultaneous and sequential sides, respectively. At Week 20, these measurements were 1.67 mm (0.62) and 1.83 mm (0.57), and at Week 32, they were 1.82 mm (0.44) and 1.80 mm (0.38), respectively. GEE analysis indicated significant changes over time (*p* < 0.001); however, no significant differences were observed between the treatments (*p* = 0.705) or treatment‐time interactions (*p* = 0.592) (Figures 5 and 6).

**FIGURE 5:**
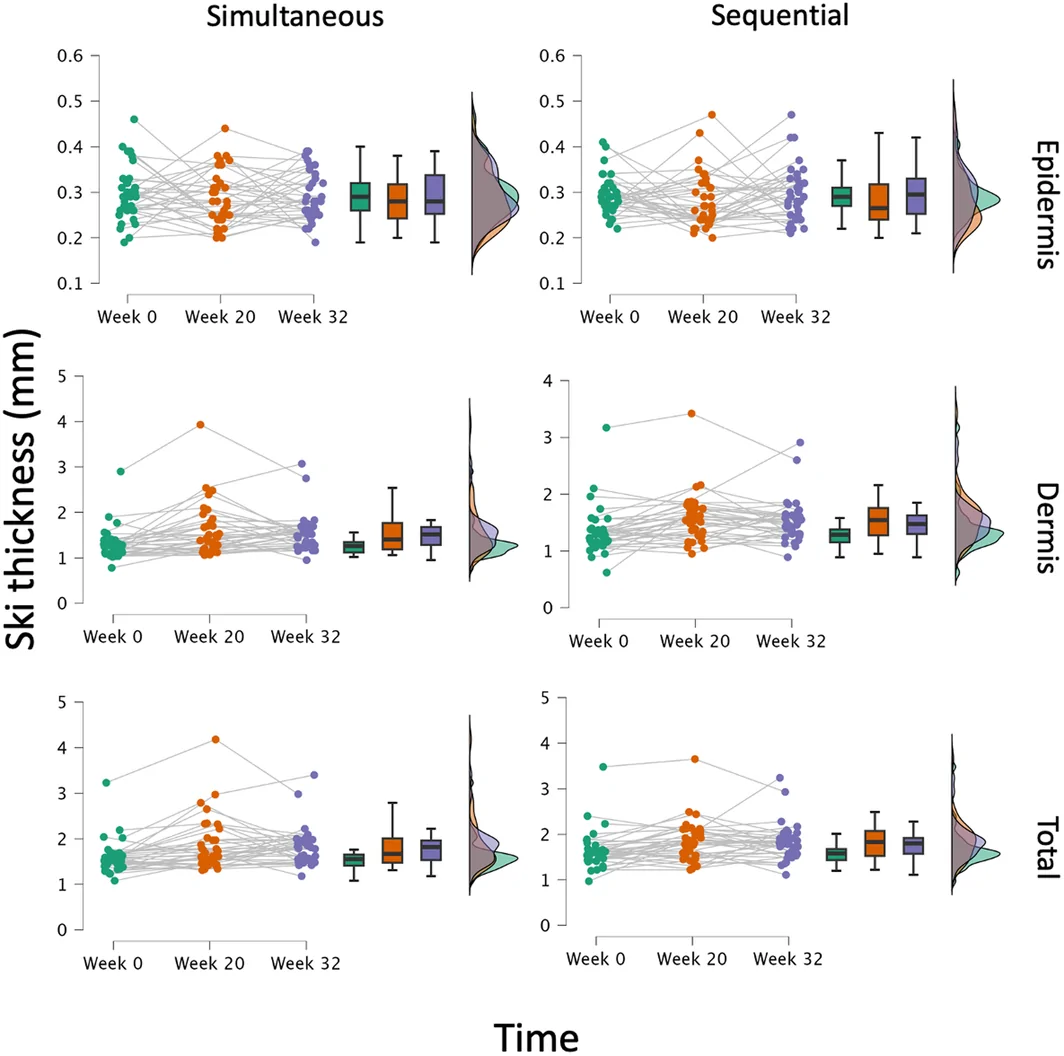
Longitudinal assessment of skin thickness by treatment.

**FIGURE 6:**
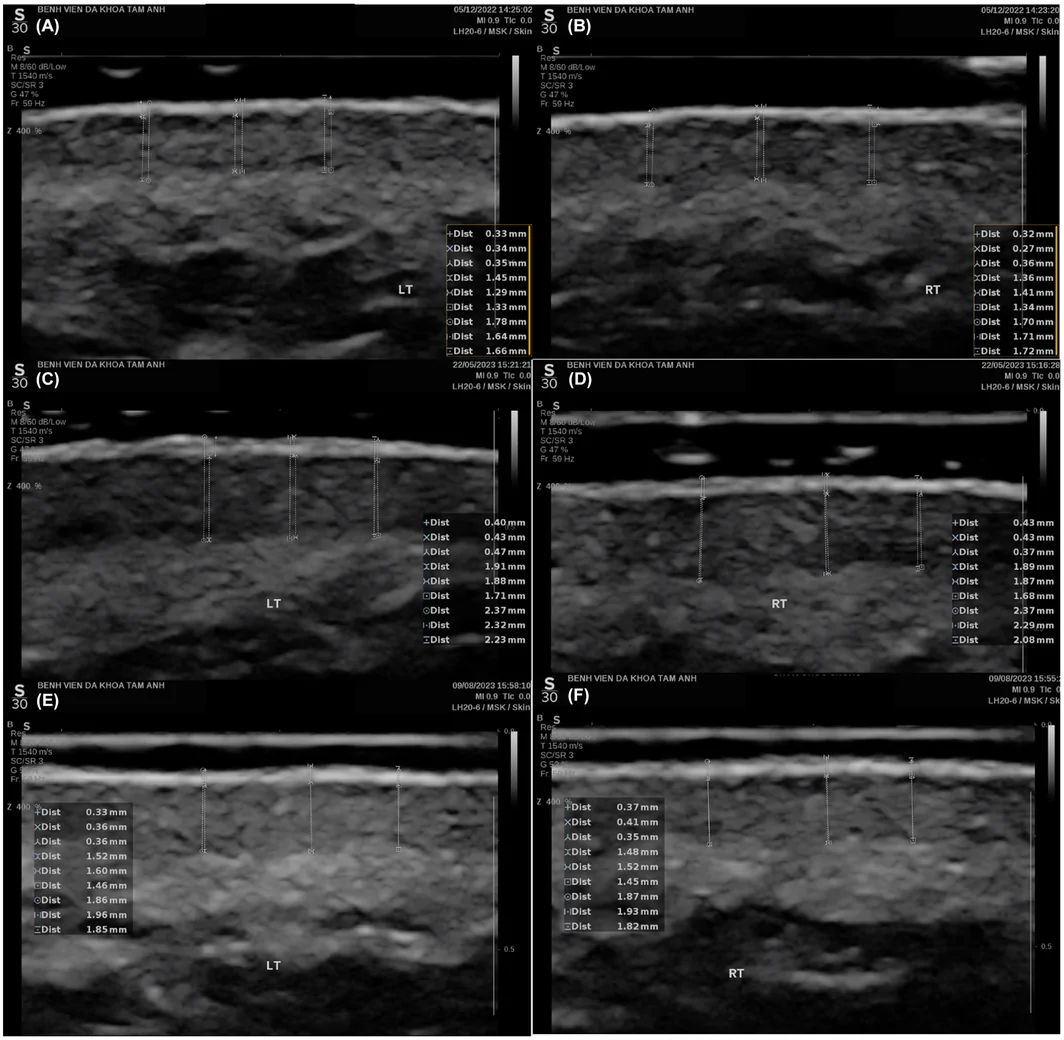
High‐frequency ultrasound assessing skin thickness. (A) Left side before treatment. (B) Right side before treatment. (C) Left side 3 months after last session. (D) Right side 3 months after last session. (E) Left side 6 months after last session. (F) Right side 6 months after last session.

### 3.5 Adverse events

Both sides showed similar erythema durations, with a median of 4 (4) days (*p* = 0.298), indicating comparable safety regarding this adverse event. However, the median duration of swelling was significantly different, being 4 (4) days on the simultaneous treatment side and 3 (2) days on the sequential treatment side (*p* < 0.001). The crusting duration was slightly longer on the simultaneous side (5 [3] days) than on the sequential side (4 [2] days); however, this difference was not statistically significant (*p* = 0.11). No significant difference was observed in the duration of bruising between the sides, with a median duration of 5 (4) days in both groups. Notably, the median downtime was significantly shorter on the simultaneous treatment side (7 [5] days) than on the sequential treatment side (11 [9] days, *p* < 0.001), highlighting a distinct advantage in the recovery time with simultaneous therapy (Figure 7).

**FIGURE 7:**
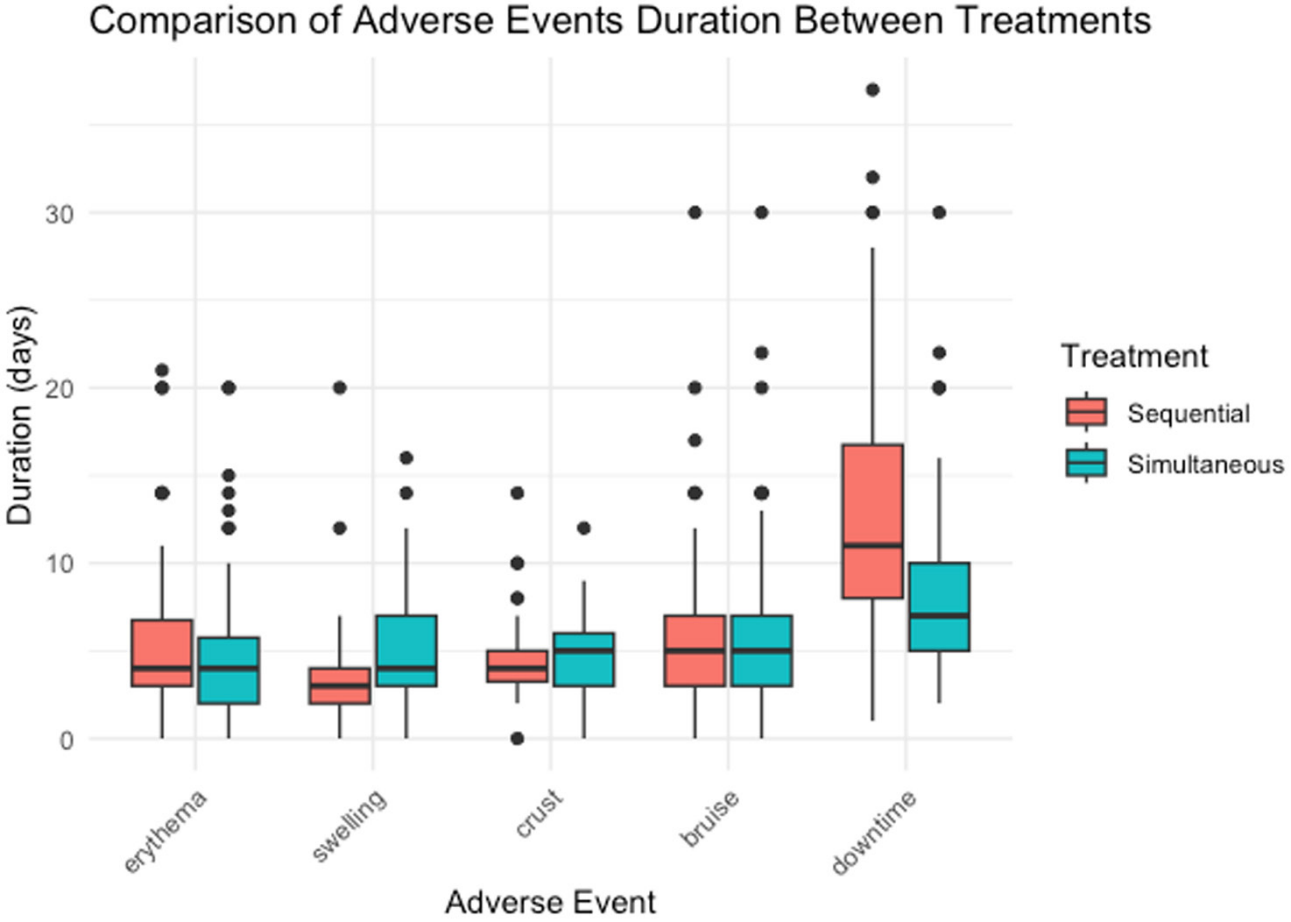
Comparison of the duration of the adverse events between sequential and simultaneous treatments.

The pain experienced during laser treatment was quantified using a 10‐point visual analog scale. On the side receiving simultaneous treatment, the median pain score was 6 (4), whereas on the side receiving sequential treatment, it was 5 (4). This difference in pain levels during laser treatment between the two sides was statistically significant (*p* = 0.002), indicating a higher pain intensity on the simultaneous treatment side. Regarding the pain experienced during subcision, the simultaneous treatment side reported a median pain score of 7 (3), compared with 6 (3) on the sequential treatment side; however, this difference was not statistically significant (*p* = 0.454), suggesting comparable pain levels during subcision between the two treatment modalities (Figure 8).

**FIGURE 8:**
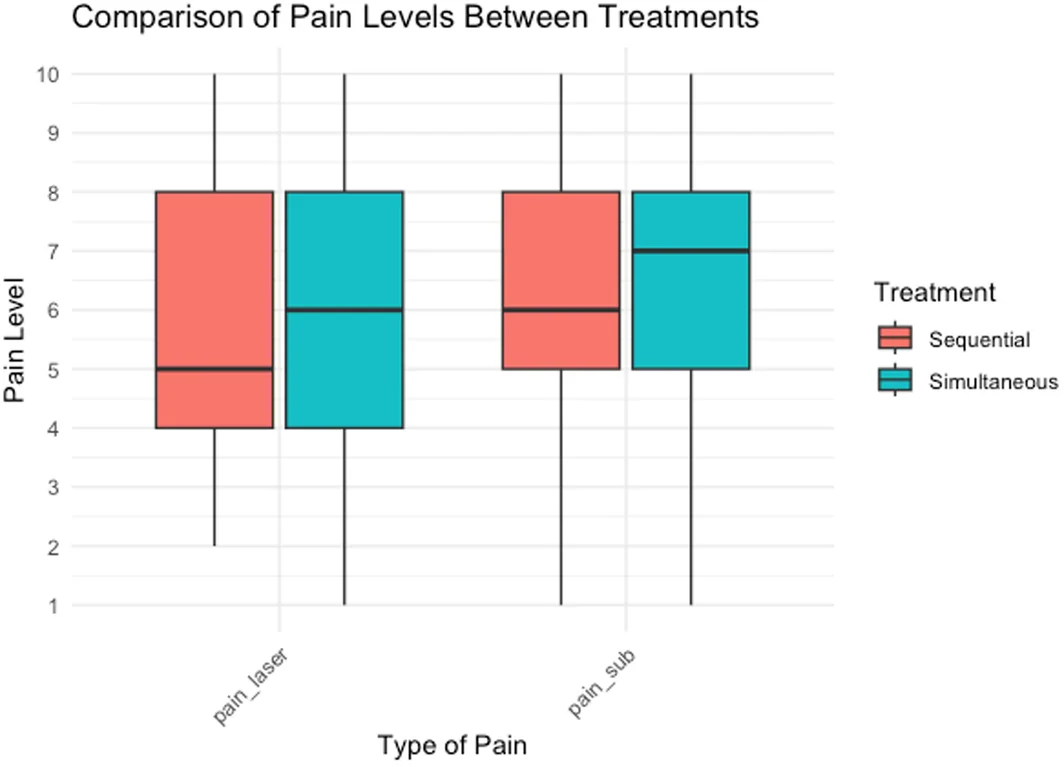
Comparison of the pain levels between two sequential and simultaneous treatments.

Upon comparing other adverse events between the simultaneous and sequential treatments, no statistically significant differences were observed in the prevalence of PIH, post‐inflammatory hypopigmentation, and acne. Regarding the presence of lumps upon evaluation over a period exceeding 5 months, no significant difference was observed in the duration between the two methods (*p* = 1.000), indicating similar resolution times across treatments (Table 2).

**TABLE 2:** Comparison of adverse events between sequential and simultaneous treatments.

	Simultaneous treatment side (*N* = 34)	Sequential treatment side (*N* = 34)	*p*‐value
Post‐inflammatory hyperpigmentation, *n* (%)	22 (64.7)	18 (52.9)	0.344^1^
Post‐inflammatory hypopigmentation, *n* (%)	8 (23.5)	5 (14.7)	0.453^1^
Acne, *n* (%)	11 (32.4)	8 (23.5)	0.453^1^
Lump self‐resolving time (months), median (IQR)	2 (0–5)	2 (0–5.25)	1.000^2^
Categories of lump resolving time, *n* (%)			
Without lumps (*N* = 23)	12 (35.3)	11 (32.4)	
With lumps (*N* = 25)	22 (64.7)	23 (67.6)	
1 month	4 (11.8)	4 (11.8)	
2 months	4 (11.8)	4 (11.8)	
3 months	2 (5.9)	4 (11.8)	
4 months	2 (5.9)	1 (2.9)	
5 months	3 (8.8)	2 (5.9)	
>5 months	7 (20.6)	8 (23.5)	

*Note*: *p*‐value extracted from ^1^McNemar's test, and ^2^Sign test.

Abbreviation: IQR, interquartile range.

## 4 DISCUSSION

Atrophic acne scars are a prevalent cosmetic concern that significantly affect the patients' quality of life imposing a psychosocial burden. Traditional monotherapies often fail to meet patient expectations, necessitating a multimodal approach to optimize therapeutic outcomes. Our study fills a critical data gap by investigating the timing of combined modalities, specifically fractional CO_2_ laser and subcision, in a split‐face comparison study design to evaluate treatment efficacy and safety, and patient satisfaction. Our study found that both simultaneous and sequential combinations of fractional CO_2_ laser and subcision were effective in improving the appearance of atrophic acne scars, as assessed by global evaluation, ECCA scoring, and high‐frequency ultrasound measurement of skin thickness, while no significant difference was observed in the efficacy between the two treatment protocols. This suggests that the choice between simultaneous and sequential combinations can be made based on individual patient needs and preferences without compromising the therapeutic benefit; patient satisfaction was similarly high on both treatment sides, reflecting the positive impact of both treatment protocols on the patient perceptions of scar appearance and treatment experience. Considering the adverse effects, although the simultaneous treatment side experienced longer durations of swelling and more intense pain during the laser procedure, the downtime was significantly shorter than that of the sequential treatment side, suggesting a trade‐off between immediate post‐procedural discomfort and downtime. Thus, reduced recovery time may be considered a practical advantage for employing simultaneous CO_2_ fractional laser treatment in patients who wish to quickly resume their daily activities.

Despite no statistically significant differences in the treatment efficacy or patient satisfaction between the two sides, both treatment combinations achieved high efficacy in our study. This finding aligns with that of previous studies on treatment of acne scars with a combination of fractional laser and subcision.^6^, ^7^, ^8^, ^9^, ^10^ The majority of patients in our study reported a satisfaction level of “satisfied,” which is similar to the outcomes of previous studies.^7^, ^10^ Treatment efficacy in our study was assessed using a quartile scale at 3 and 6 months after the final treatment session. The predominant improvement levels were “moderate” and “significant,” with a minority of cases reporting a “marked” improvement level in both treatment modalities. We observed certain similarities upon comparing our findings with those of other studies. Nilforoushzadeh et al. reported an average improvement of 54.7%, corresponding to a “significant” improvement on a quartile scale.^6^ This level of efficacy aligns closely with the outcomes of the present study. Taylor et al. reported an average improvement score of 2.9 on a 4‐point scale, indicating a “moderate” improvement, similar to the results of our study.^9^ Certain studies have reported higher improvement rates than our findings. Lee reported improvement levels of “mild” (7%), “moderate” (29%), “significant” (57%), and “marked” (7%), with a higher proportion of “significant” improvement compared to our study.^8^ However, our study also reported a higher “marked” improvement rate, which was higher than the outcomes reported in Lee's study. Despite the similar number of treatment sessions, the earlier assessment of treatment efficacy (2 months after the last treatment session) in Lee's study may suggest that their treatment protocol yielded “significant” improvement more promptly. The study conducted by Anupama et al. demonstrated “mild” (13.4%), “significant” (30.43%), and “marked” (56.52%) improvements, which are considerably higher than those reported in our study and other studies.^7^ However, this study employed the Goodman and Baron scale to classify the improvement levels. Owing to the use of a different methodological approach, a direct comparison of the results with those of other studies may not be feasible. The study by Ibrahim et al. reported improvement levels in the side treated with combined fractional CO_2_ laser and scar subcision as “mild” (20%), “moderate” (40%), “significant” (10%), and “marked” (0%)^10^; both combined treatment approaches in our study demonstrated higher rates of “significant” and “marked” improvement. The similarity in the timing of efficacy assessment (3 months posttreatment) and treatment protocol (three treatment sessions with one‐month intervals) between our study and Ibrahim's study ensures a direct comparison.

We observed a trend of gradual improvement in the atrophic acne scars in each of the three treatment sessions, which continued to progress favorably for up to 3 months. This consistent improvement aligns with the expectations concerning the therapeutic effects of the fractional CO_2_ laser and subcision interventions. However, although statistically insignificant, a slight reduction was observed in the improvement levels at the 6‐month follow‐up on both the simultaneous and sequential sides. This phenomenon has been sparsely documented, with Elcin et al. reporting a decrease in the scar appearance at a long‐term follow‐up of 3 years compared with short‐term results at 3 months.^11^ Possible explanations for the reduction in scar quality over time include the resolution of mild swelling post‐treatment, which initially renders scars less conspicuous. As the swelling subsided completely by the 6‐month evaluation, some scar depressions may have reappeared. Additionally, an adverse event of subcision is the development of subcutaneous lumps, which may unintentionally act as a natural filler, resulting in temporary improvement in the appearance of the scar. As these lumps resolve, the scars may become more apparent again, contributing to a decrease in the treatment effect.

To our knowledge, few studies have used HFUS to measure the changes in skin thickness after treatment of atrophic acne scars with fractional lasers. However, HFUS has not been previously applied to this specific treatment combination. HFUS provides information about skin thickness changes at baseline, 3 months, and 6 months after treatment. No significant changes in the epidermal thickness were observed over time or between the treatment modalities, suggesting that the primary targets of these treatments may lie within the deeper skin layers. This outcome differs from those of studies that used fractional laser therapy alone or in combination with other methods. Nilforoushzadeh et al. reported a significant increase in the epidermal thickness when treating acne scars with subcision followed by autologous fibroblast injections, from 182.40 to 290.19 μm (*p* < 0.001).^12^ This significant thickening could be attributed to the regenerative stimulation induced by the injected fibroblasts affecting all the skin layers. Our study focused on the dermal effects of combining subcision with a fractional CO_2_ laser, mainly targeting the dermal tissue. A study by Malinowska reported a statistically significant reduction in the epidermal thickness after treating acne scars with three monthly sessions of erbium‐doped yttrium aluminium garnet (Er:YAG) laser, from 0.13 to 0.11 mm (*p* < 0.001).^13^ This difference could be explained by the ablation mechanism of the lasers used. The Er:YAG laser, with a wavelength of 2940 nm, achieves more vertical ablation by directly vaporizing the tissue, whereas the CO_2_ laser, at 10600 nm, exerts a more horizontal effect through photothermal ablation, leading to a broader thermal damage zone.

Significant improvements were observed in the dermal and total skin thicknesses following simultaneous and sequential treatments for atrophic acne scars. An increase in the dermal thickness was observed from baseline to Week 20, which remained elevated until Week 32. GEE analysis revealed significant changes over time (*p* < 0.001) on both sides. These findings suggest that both combined treatment modalities effectively enhanced dermal thickness, implying successful collagen synthesis and scar improvement. Malinowska et al. reported an insignificant increase in the dermal thickness after Er:YAG laser treatment (from 1.22 to 1.23 mm, *p* = 0.327).^13^ This could be attributed to the differences in the thermal damage mechanisms of the Er:YAG and CO_2_ lasers. This insignificant improvement may also be explained by the timing of the 1‐month assessment in Malinowska's study. Our results demonstrated a significant increase in the total skin thickness over time (*p* < 0.001); however, no significant differences were observed between the treatment modalities. This underscores the equivalence of the combined treatments in improving skin thickness. However, the extent of improvement was less than that reported by Nilforoushzadeh et al., who combined subcision with autologous fibroblast injections, a treatment likely to optimize regenerative processes, resulting in considerable thickening of the skin.^12^

Our study demonstrated differences in the adverse events, which included pain during laser treatment, duration of swelling, and downtime, between the two treatment modalities. Other adverse events, such as pain during subcision, crusting, bruising, PIH, post‐inflammatory hypopigmentation, subcutaneous lump, and acne, were comparable between the two treatment combinations, which is consistent with the results of previous studies.^6^, ^7^, ^11^ Although statistically insignificant, the slight variation in crusting duration might be attributed to the inflammatory response after treatment on the simultaneous and sequential sides. In simultaneous treatment, the inflammatory response leads to enhanced blood flow to the area treated with subcision combined with potential subcutaneous bleeding after subcision may contribute to a more sustained crust formation in MTZs after treated with fractional CO_2_ laser. To evaluate the pain experienced during laser treatment, we used a 10‐point visual analog scale, and patients reported experiencing pain of moderate intensity on both treatment sides. However, the side receiving simultaneous treatment demonstrated a significantly higher pain level than that receiving sequential treatment (*p* = 0.002). This disparity could be attributed to a reduction in the effect of local anesthesia over time after subcision, suggesting that simultaneous treatment may provide a more comfortable patient experience. Swelling was another adverse event that yielded significantly different results between the two sides (*p* < 0.001), with the simultaneous treatment side experiencing a median of 4 days of swelling compared with 3 days on the sequential side. Prolonged swelling may be attributed to an inflammatory response triggered by both subcision and laser treatment, which becomes more pronounced when both procedures are performed simultaneously. A notable difference was observed in the downtime, defined as the total time that patients experienced visible side effects that affected their personal and social lives. The simultaneous treatment side had a shorter median downtime of 7 days compared with 11 days on the sequential treatment side (*p* < 0.001). This finding suggests that simultaneous treatment may be preferable for patients who are concerned about the impact of treatment‐related side effects on their daily lives, thereby offering a quicker return to normal activities. In summary, while some adverse events were similar between both combination modalities and consistent with previous literature, differences in pain during laser treatment, swelling, and particularly, downtime offer valuable insights for clinical decision‐making. These findings emphasize the importance of considering patient preferences and the practical implications of treatment downtime when selecting the most appropriate combination therapy for acne scars.

The present findings should be interpreted with caution owing to the presence of several limitations to the study. An essential strength of this study was that the results were derived from a split‐face trial, allowing each case to serve as its control. However, our study design lacked randomization; thus, a possibility of selection bias and presence of confounding factors exists that could affect the accuracy and validity of our findings. Second, the sample size of 34 patients may require more power to detect significant differences between the two treatment groups, especially if the effect size is small. In addition, our convenience sampling method may not fully represent the target population, thereby limiting the generalizability of our results. Blinding was also challenging in our study owing to the nature of the treatments, which could introduce bias in both treatment administration and patient‐reported outcomes. Importantly, we did not consider confounding variables that could influence the treatment outcomes, which is another limitation.

In conclusion, fractional CO_2_ laser combined with subcision simultaneously or sequentially improved the appearance of atrophic acne scars without significantly impacting the efficacy between the two modalities. Downtime, pain experienced during laser treatment, and duration of swelling are factors that should be considered when determining the most suitable treatment option for individual patients. These aspects highlight the necessity for a tailored approach that considers patient preferences and the capacity to manage post‐treatment recovery.

## AUTHOR CONTRIBUTIONS

This manuscript is a collaborative effort involving four authors, each contributing significantly to the presented research. Bao Quoc Tran: Protocol development, data acquisition, data analysis, and manuscript edit and revision. Tu Nguyen Anh Tran: Data acquisition, data analysis, and manuscript edit and revision. Em Van Loi Doan: Data acquisition, data analysis, and manuscript edit and revision. Thuy Thi Phan Nguyen: Data acquisition, data analysis, and manuscript edit and revision. Hao Trong Nguyen: Protocol development, data acquisition, data analysis, and manuscript edit and revision.

## FUNDING INFORMATION

This study was supported by the Ho Chi Minh City Hospital of Dermato‐Venereology.

## CONFLICT OF INTEREST STATEMENT

The authors declare no conflicts of interest regarding the publication of this manuscript.

## ETHICS STATEMENT

This study was approved by the Ethics Committee of the Ho Chi Minh City Hospital of Dermato‐Venereology (certificate number 1607/CN‐BVDL; December 1, 2022). Written informed consent was obtained after informing the participants of the detailed study procedures. This study complied with the tenets of the Declaration of Helsinki and was registered at ClinicalTrials.gov: NCT05688202 (https://classic.clinicaltrials.gov/ct2/show/NCT05688202).

## CONSENT

In compliance with ethical standards and privacy concerns, all identifiable photographs in this manuscript have received explicit consent from the individuals depicted for the publication of the photos. Consent has been obtained using the “Patient Consent Form” as prescribed by Wiley, written in English and Vietnamese. This consent form encompasses the agreement of individuals for their images to be used in this publication and for any related scholarly purposes, ensuring complete understanding and agreement on the part of the participants regarding the use, scope, and implications of sharing their images in a public academic forum.

## Data Availability

The data that support the findings of this study are available from the corresponding author upon reasonable request.
